# Supplementary material for: Longitudinal transcriptional changes reveal genes from the natural killer cell-mediated cytotoxicity pathway as critical players underlying COVID-19 progression
Source: eLife. 2024 Oct 29;13:RP94242. doi: 10.7554/eLife.94242 (PMC11521369; doi:10.7554/eLife.94242)
Supplement: Figure 3—source data 2. [file elife-94242-fig3-data2.docx]

**Figure 3-source data 2**. Enriched pathways from genes upregulated in severe COVID-19 patients by longitudinal analysis for figures 3B, D, F.

| **SEVERE - DAY 0** | |
| --- | --- |
| Neutrophil extracellular trap formation | H2BC7, CR1, H2BC6, AQP9, NCF4, C5AR1, FPR1, FPR2, MAPK14, MPO, TLR8, PADI4, H4C4, FCGR1A, ELANE, TLR2 |
| Hematopoietic cell lineage | GYPA, CSF3R, IL4R, CR1, MME, IL1R1, FLT3, IL1R2, FCGR1A, CD55 |
| Viral protein interaction with cytokine and cytokine receptor | IL18RAP, TNFSF14, CXCR1, TNFRSF10C, CXCL1, CXCL2, IL18R1 |
| Amoebiasis | SERPINB10, IL1R1, ARG1, IL1R2, FN1, CXCL1, CXCL2, TLR2 |
| Complement and coagulation cascades | THBD, CR1, SERPINB2, PLAU, C5AR1, VSIG4, CD55, F5 |
|  |  |
| **SEVERE - DAY 7** | |
| Transcriptional misregulation in cancer | CEBPB, FLT1, BCL2A1, GADD45A, FLT3, CEBPE, IL1R2, DEFA4, ZBTB16, DEFA3, MPO, MMP9, GADD45G, BCL6, PLAU, PPARG, ERG, FCGR1A, ELANE |
| Neutrophil extracellular trap formation | CR1, AQP9, NCF4, C5AR1, FPR1, FPR2, AZU1, MAPK14, MPO, TLR8, CTSG, PADI4, FCGR1A, ELANE, CAMP, TLR2 |
| IL-17 signaling pathway | FOSL1, CXCL6, CEBPB, LCN2, TNFAIP3, CXCL1, MAPK14, CXCL2, MMP9, S100A9, S100A8 |
| TNF signaling pathway | SOCS3, CXCL6, CEBPB, TNFAIP3, CXCL1, MAPK14, CXCL2, MMP9, IL18R1, CREB5 |
| Hematopoietic cell lineage | GYPA, CSF3R, IL4R, CR1, MME, IL1R1, FLT3, IL1R2, CD24, FCGR1A, CD55 |
|  |  |
| **SEVERE - DAY 28** | |
| Malaria | CR1, TLR9, SDC1, THBS1, TLR2 |
| Legionellosis | CR1, TLR5, CXCL2, TLR2, HSPA1A |
| Neutrophil extracellular trap formation | CR1, C5AR1, FPR1, TLR8, FPR2, FCGR1A, MAPK14, TLR2 |
| Toll-like receptor signaling pathway | CXCL10, TLR9, TLR8, TLR5, MAPK14, TLR2 |
| Complement and coagulation cascades | THBD, CR1, SERPINB2, C5AR1, CD55 |
