# Supplementary material for: Longitudinal transcriptional changes reveal genes from the natural killer cell-mediated cytotoxicity pathway as critical players underlying COVID-19 progression
Source: eLife. 2024 Oct 29;13:RP94242. doi: 10.7554/eLife.94242 (PMC11521369; doi:10.7554/eLife.94242)

A Day 0 - KEGG graph of Natural Killer cell mediated cytotoxicity pathway

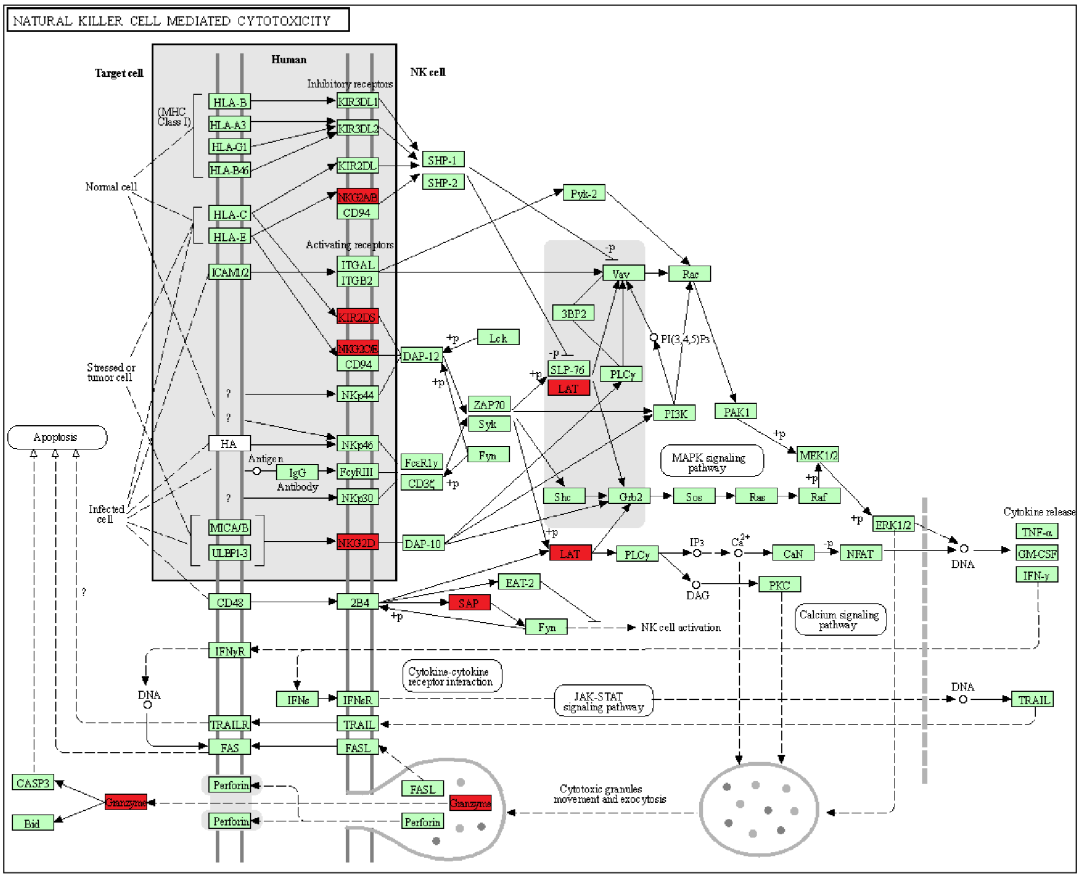

B Day 7 - KEGG graph of Natural Killer cell mediated cytotoxicity pathway

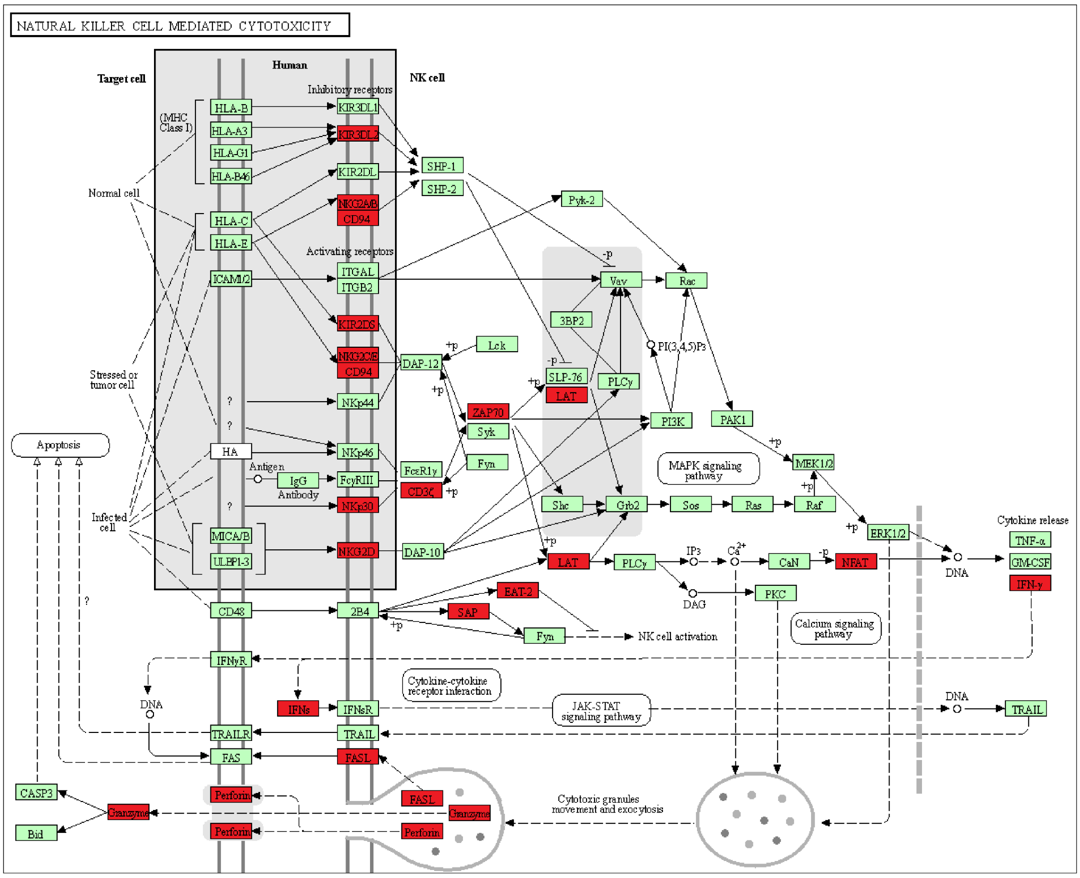

C Day 0 - KEGG graph of Th1 and Th2 cell differentiation

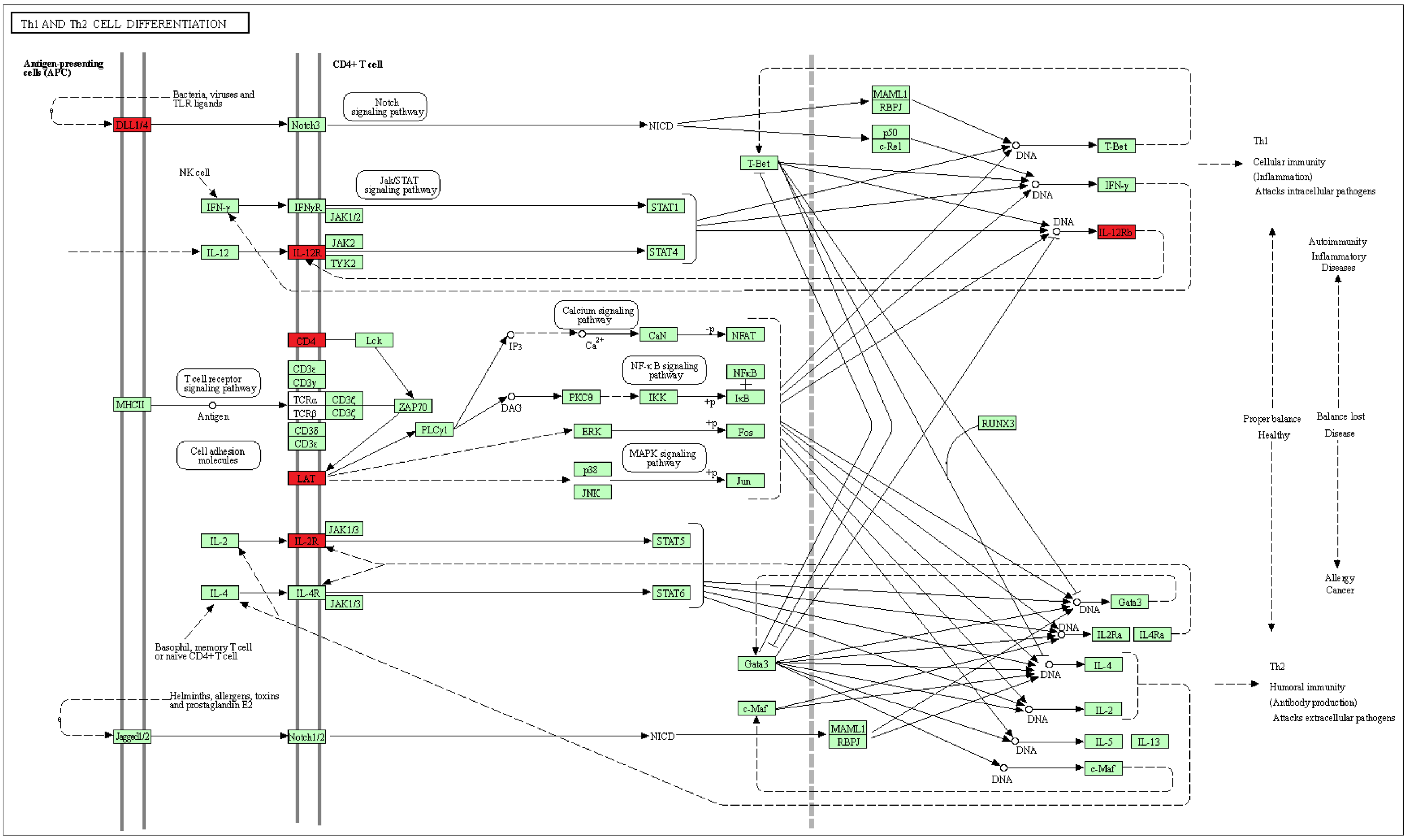

D Day 7 - KEGG graph of Th1 and Th2 cell differentiation

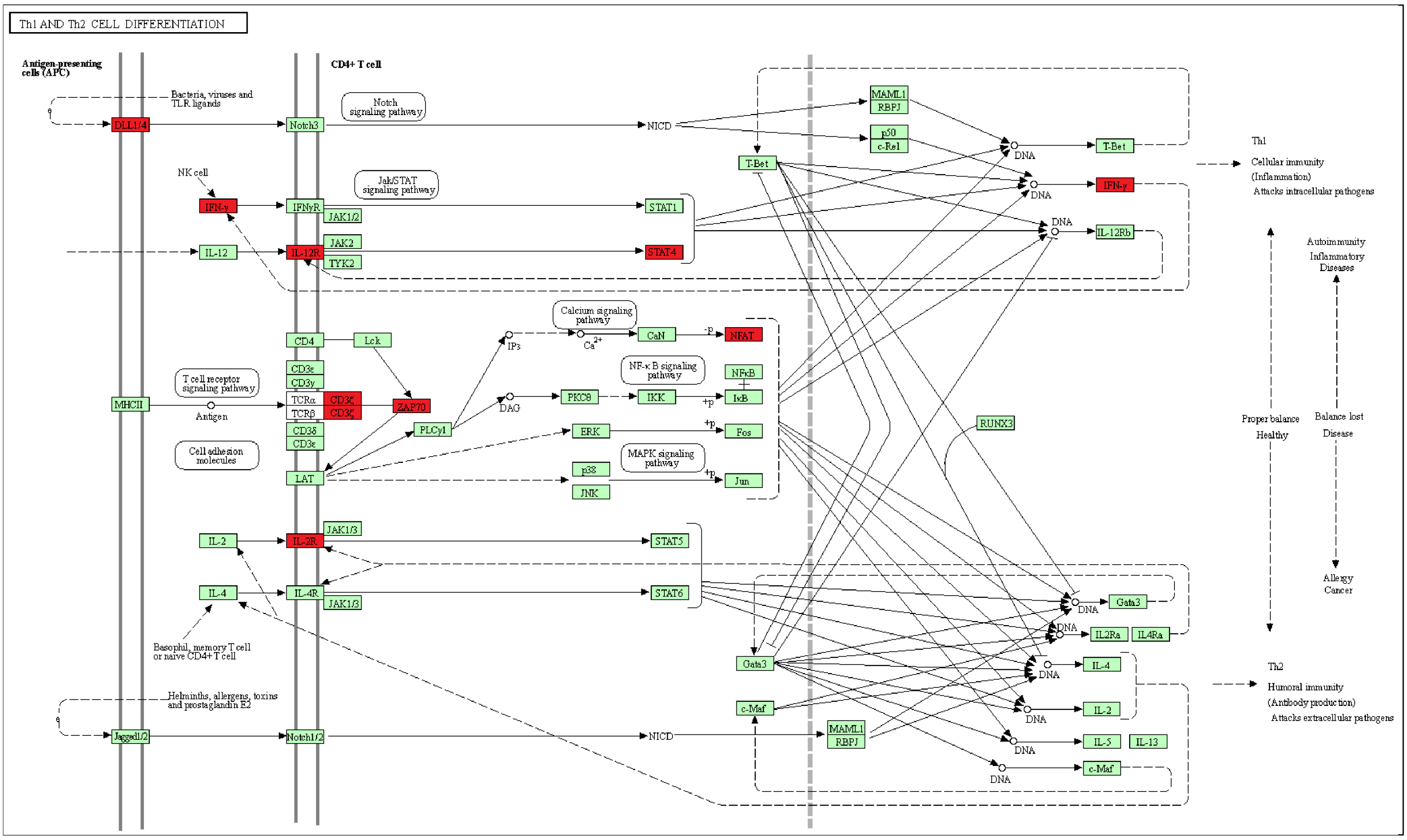

E Day 0 - KEGG graph of Cytokine-cytokine receptor interaction

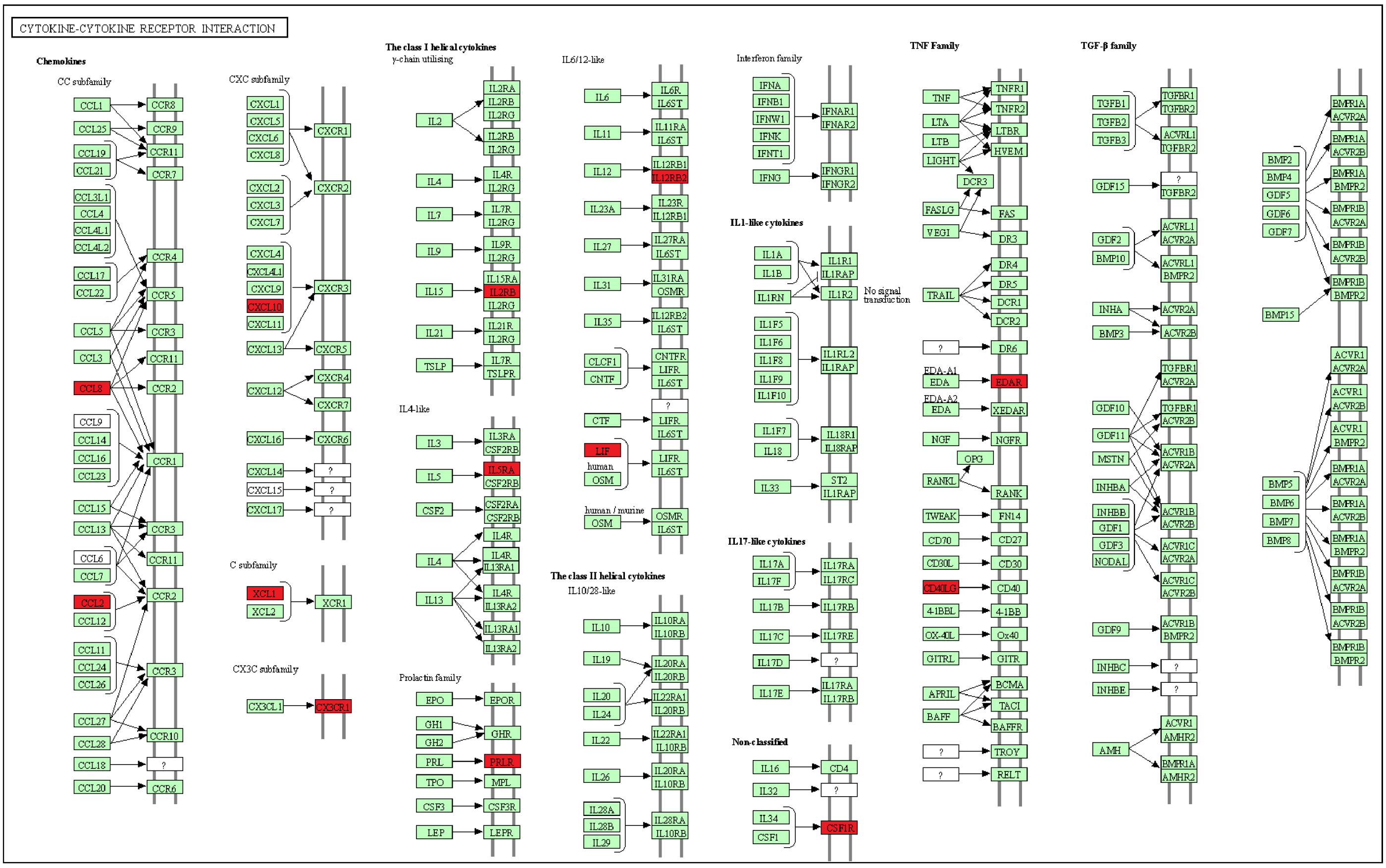

Supplement: Figure 4—source data 1. — Red boxes depict upregulated genes, whereas green boxes depict genes without significant differential gene expression within each KEGG pathway. [file elife-94242-fig4-data1.pdf]
